# Complete genome sequence data describing the biosynthetic potential of kanosamine and bacilysin in *Bacillus subtilis* AMFE023/7 from Thai fermented fish (Pla Ra)

**DOI:** 10.1016/j.dib.2025.111860

**Published:** 2025-07-09

**Authors:** Montri Yasawong, Thunwarat Songngamsuk, Manassanan Phatcharaharikarn, Parweenuch Santaweesuk, Prapimpun Wongchitrat, Prasit Palittapongarnpim, Wongwarut Boonyanugomol, Arunee Thong-on, Sasalux Kaewbutra, Kamolchanok Rukseree

**Affiliations:** a Programme on Environmental Toxicology, Chulabhorn Graduate Institute, Bangkok 10210, Thailand; b Center of Excellence on Environmental Health and Toxicology (EHT), OPS, MHESI, Bangkok 10400, Thailand; c Center for Research Innovation and Biomedical Informatics, Faculty of Medical Technology, Mahidol University, Nakhon Pathom 73170, Thailand; d Pornchai Matangkasombut Center for Microbial Genomics, Department of Microbiology, Faculty of Science, Mahidol University, Bangkok 10400, Thailand; e Unit of Water and Food Analysis, Department of Medical Science, Amnatcharoen Campus, Mahidol University, Amnatcharoen 37000, Thailand; f Department of Medical Science, Amnatcharoen Campus, Mahidol University, Amnatcharoen 37000, Thailand

**Keywords:** Bacillibactin, Bacilysin, Fengycin, Fermented fish, Kanosamine, Surfactin

## Abstract

*Bacillus subtilis* AMFE023/7, a Gram-positive bacterium with aciditolerans and halotolerant properties, was isolated from Pla Ra, a traditional fermented fish product popular in Thai cuisine. The sample was obtained from a local morning market in Amnat Charoen, Thailand. The complete genome was assembled through the integration of complementary sequencing platforms, specifically Illumina and PacBio. The genome spans 4132,147 base pairs with a GC content of 43.87 %. Comparison with the reference strain *B. subtilis* ATCC 6051^T^ using digital DNA-DNA hybridisation confirmed the identity of the isolate with 89.3 % similarity. The genome analysis revealed gene clusters responsible for producing potentially useful compounds such as kanosamine and bacilysin, which have antimicrobial properties. This genome sequence provides valuable data for researchers studying microbial communities in fermented foods, those conducting comparative genomics, and scientists searching for new bioactive compounds. The complete genome sequence data has been made publicly available through NCBI under BioProject accession number PRJNA1253417.

Specifications Table

**Table:** 

Subject	Genomics
Specific subject area	Complete genome sequencing and annotation of *Bacillus* for bioactive compound screening
Data format	Raw and analysed
Type of data	Tables, figures
Data collection	DNA was extracted using the Quick-DNA HMW MagBead Kit and sequenced using BGISEQ-500 and PacBio Revio. The reads were processed using fastp v0.23.4 and fastplong v0.2.0, and the hybrid assemblies were then assembled using Unicycler v0.5.1. The quality assessment was performed using QUAST v5.2.0 and CheckM2 v1.0.1. Genome mapping was performed using Bakta v1.10.4, and taxonomic identification was performed using TYGS for dDDH analysis. NCBI PGAP annotated the genome, with KAAS analysing metabolic pathways and antiSMASH v7.1.0 identifying secondary metabolites and biosynthetic gene clusters.
Data source location	*B. subtilis* AMFE023/7 was isolated from Thai fermented fish (Pla Ra) collected from Amnat Charoen Morning Market, Thailand (15°51′36.9″N, 104°37′39.0″E).
Data accessibility	Data Identification number: BioProject: PRJNA1253417 BioSample: SAMN48076195 Direct URL to data: https://www.ncbi.nlm.nih.gov/biosample/?term=SAMN48076195
Related research articles	None

## 1 Value of the Data

• The complete genome sequence of *B. subtilis* AMFE023/7 isolated from high-salinity (15.84 % w/v) and acidic (pH 5.58) fermented fish environments provides data for studying microbial adaptation to extreme conditions.
• This genome reveals the complete kanosamine and bacilysin biosynthetic pathways of *B. subtilis* and contributes to our understanding of antimicrobial compound production in *B. subtilis* strains from fermented foods.
• The sequence data enable comparative analyses with other *Bacillus* species and support the identification of biosynthetic gene clusters for bioactive compound research.

## 2 Data Description

**Fig. 1:**
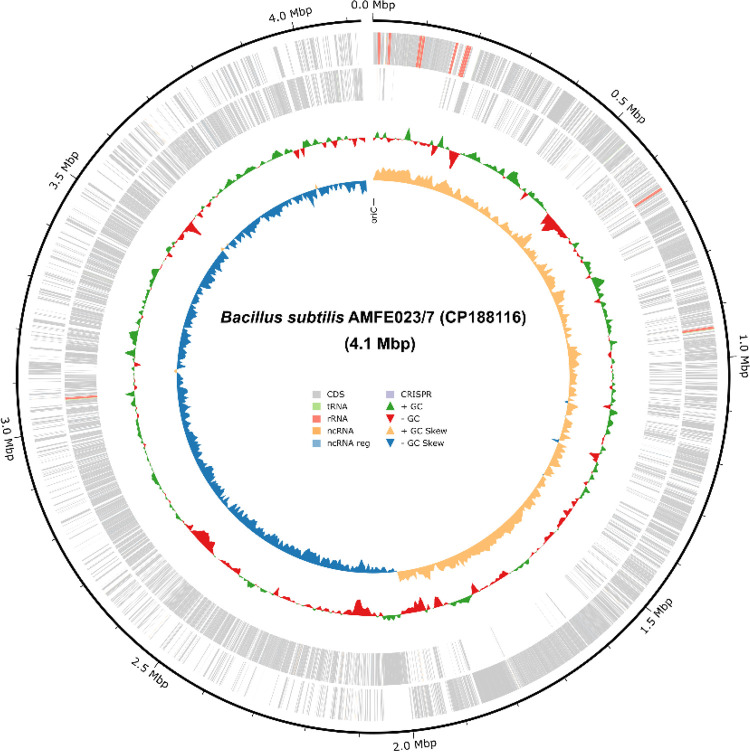
Circular genome map of *B. subtilis* AMFE023/7. This representation of the 4.1-Mbp genome shows the position markers in Mbp around the outer circle. The rings display, from outside inwards, the coding sequences (CDS in light grey) and CRISPR regions (in light purple), followed by RNA genes (tRNA in lime green, rRNA in coral, ncRNA in peach with regulatory elements in sky blue). The inner rings illustrate the GC patterns, with green and red triangles indicating variations in the GC content, and light orange and blue triangles represent the GC skew direction.

Here, we present the complete genome sequence data of *B. subtilis* AMFE023/7 (CP188116) (Fig. 1), including analysis of gene clusters and secondary metabolite biosynthetic potential.

The complete genome of *B. subtilis* AMFE023/7 (CP188116) comprises 4125,200 bp with a GC content of 43.79 % and 727× coverage. The analysis confirmed 100 % genome completeness with minimal contamination (<1 %), indicating a high-quality assembly (Table 1).

**Table 1:** Genomic features and assembly statistics of *B. subtilis* AMFE023/7.

Attribute	*B. subtilis* AMFE023/7
Genome size (bp)	4125,200
Completeness (%)	100
Contamination (%)	0.03
Genome coverage	727×
GC content (%)	43.79
Total gene	4287
Total CDS	4166
Genes (coding)	4057
tRNA	86
rRNA	30
ncRNA	5

**Fig. 2:**
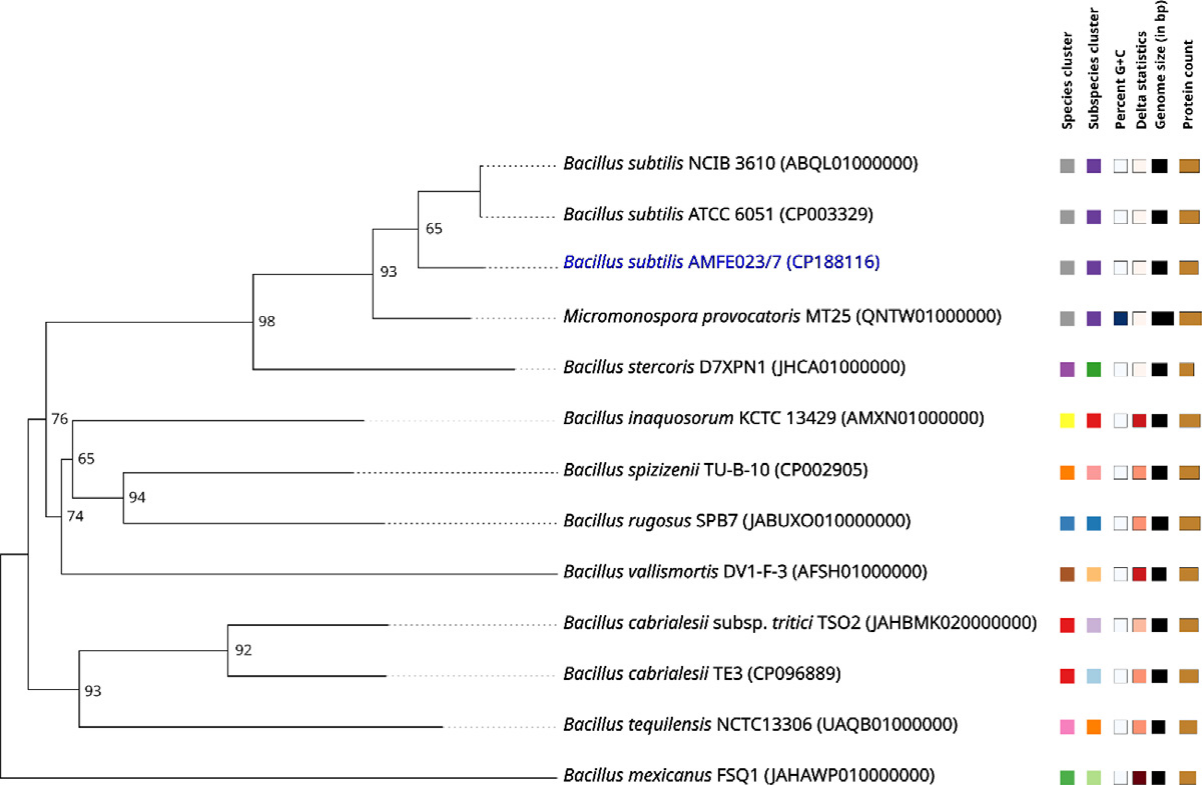
Phylogenomic tree reconstructed from whole-genome sequence data of *B. subtilis* AMFE023/7 and closely related type strains using the TYGS platform. Branch support values represent pseudo-bootstrap percentages greater than 70 % from 100 replicates generated via Genome BLAST Distance Phylogeny (GBDP), with a mean branch support of 93.6 %.

Genomic relatedness assessment through digital DNA-DNA hybridisation (dDDH) revealed a value of 89.3 % between strain AMFE023/7 and the type strain *B. subtilis* ATCC 6051^T^, substantially exceeding the 70 % threshold for species delineation [1] and thereby confirming the taxonomic assignment of AMFE023/7 as a *B. subtilis* species. The evolutionary relationships between AMFE023/7 and phylogenetically adjacent type strains are illustrated in the comprehensive phylogenomic tree presented in Fig. 2.

The genome analysis of *B. subtilis* AMFE023/7 revealed eight biosynthetic gene clusters encoding diverse bioactive compounds (Table 2). Seven clusters displayed 100 % similarity with known pathways, whereas the surfactin cluster showed 82 % similarity. This genetic architecture spans multiple compound classes, including peptides, modified peptides, polyketides, and specialised metabolites.

**Table 2:** Secondary metabolite biosynthesis gene clusters identified using antiSMASH.

Type	Position (bp)	The closest known biosynthetic class	Predicted secondary metabolites	Similarity
CDPS	3481,121–3501,867	Other	Pulcherriminic acid	100 %
Lanthipeptide class I	220,133–244,590	RiPP:Lanthipeptide	Subtilomycin	100 %
NRP-metallophore, NRPS	3149,080–3200,857	NRP	Bacillibactin	100 %
NRPS, Betalactone	1990,471–2068,218	NRP	Fengycin	100 %
Other	3752,950–3794,368	Other	Bacilysin	100 %
Sactipeptide	3728,340–3749,951	RiPP:Thiopeptide	Subtilosin A	100 %
TransAT-PKS, PKS-like, T3PKS, NRPS	1766,992–1881,741	Polyketide+NRP	Bacillaene	100 %
NRPS	368,172–433,563	NRP:Lipopeptide	Surfactin	82 %

CDPS, tRNA-dependent cyclodipeptide synthases; Lanthipeptide class I, class I lanthipeptides like nisin; NRP-metallophore, non-ribosomal peptide metallophores; Betalactone, beta-lactone containing protease inhibitor; TransAT-PKS, Trans-AT PKS; PKS-like, Other types of PKS; T3PKS, Type III PKS.

**Fig. 3:**
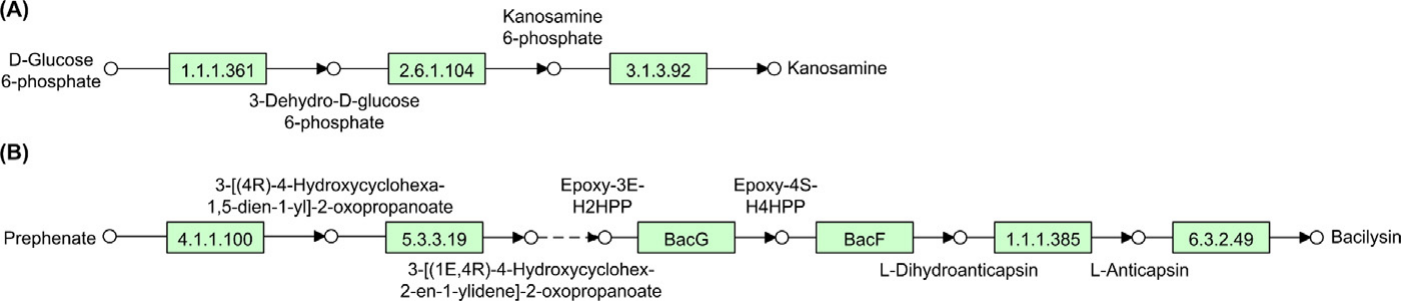
Antimicrobial biosynthetic pathways in *B. subtilis* AMFE023/7. (A) The kanosamine pathway converts D-glucose-6-phosphate through three enzymatic steps (EC 1.1.1.361, EC 2.6.1.104, and EC 3.1.3.92) to produce an aminoglycoside antimicrobial. (B) The bacilysin pathway transforms prephenate through six catalytic reactions (including EC 4.1.1.100, EC 5.3.3.19, BacG, BacF, EC 1.1.1.385, and EC 6.3.2.49) to yield a dipeptide antibiotic targeting bacterial cell wall synthesis.

*B. subtilis* AMFE023/7 may produce an impressive antimicrobial arsenal. The genome encodes the potential production of membrane-disrupting surfactin [2] and antifungal fengycin [3]. The bacteriocin repertoire includes thioether-rich subtilosin A, which is active against diverse bacteria [4] and subtilomycin targeting foodborne pathogens [5]. In addition to direct antimicrobial activity, this strain exhibits sophisticated competitive mechanisms. The genetic blueprint includes pathways for iron-scavenging bacillibactin [6] and pigment-forming pulcherriminic acid [7]. Additionally, *B. subtilis* AMFE023/7 may produce cell wall-targeting bacilysin [8,9] and protein synthesis-inhibiting bacillaene [10]. Further pathway reconstruction analysis identified complete biosynthetic routes for key antibiotics in *B. subtilis* AMFE023/7. As illustrated in Fig. 3, two key antibiotic biosynthesis pathways, kanosamine and bacilysin, were fully mapped. Although these pathways are commonly present among *B. subtilis* strains, the complete genomic characterisation and pathway reconstruction in AMFE023/7 provide detailed insights into the antimicrobial production capabilities of strains adapted to extreme fermented food environments. The kanosamine pathway (Fig. 3A) elegantly transforms the common metabolic intermediate D-glucose-6-phosphate through a precisely orchestrated three-enzyme cascade, ultimately yielding kanosamine, an aminoglycoside that serves as part of the strain’s chemical defence system against competing microorganisms. This three-enzyme cascade is encoded by the *ntdABC* gene cluster. The bacilysin pathway (Fig. 3B) is a more complex route involving multiple specialised enzymes, beginning with prephenate and proceeding through several intermediates to eventually form bacilysin, a dipeptide antibiotic that disrupts bacterial cell wall synthesis. This biosynthetic pathway is encoded by the *bacABCDEFG* gene cluster, which comprises seven genes responsible for complete dipeptide antibiotic synthesis.

This diverse metabolite profile highlights the biosynthetic potential of microorganisms isolated from traditional fermented foods. Although the high pathway similarities suggest functional capacity for bioactive compound production, it should be noted that experimental validation would be necessary to confirm actual production. *B. subtilis* AMFE023/7 represents not only a component of traditional fermented fish microbial communities but also a potential source of bioactive compounds. Further investigations are required to elucidate the potential practical applications of these compounds across diverse fields.

## 3 Experimental Design, Materials, and Methods

### 3.1 Bacterial isolation

Strain AMFE023/7 was isolated from Pla Ra (fermented fish) from Amnat Charoen Morning Market, Thailand (15°51′36.9″N, 104°37′39.0″E). At the time of collection, the sample exhibited an acidic pH of 5.58 and high salinity of 15.84 % (w/v), characteristic of traditional fermented fish preparations. Bacterial isolation was performed by serial dilution plating. One millilitre of fermented fish sample was diluted in nine millilitres of 0.1 % peptone water, and 100 microlitres aliquots were spread onto Luria-Bertani (LB) agar plates and incubated overnight at 37 °C. The bacterial isolates were selected for their strong proteolytic activity on skim milk agar and fibrinolytic capacity on fibrin plates [11]. The isolate producing clear zones was collected and stored in LB broth containing 20 % glycerol at −20 °C for further study.

### 3.2 Genomic DNA preparation

Genomic DNA was extracted from *B. subtilis* AMFE023/7 cultures grown overnight in LB broth at 30 °C with shaking at 150 rpm. The extraction protocol used a Quick-DNA HMW MagBead Kit (Zymo, USA), which effectively preserved the integrity of the bacterial chromosome. The resulting DNA preparation underwent quality assessment using Qubit fluorometric quantification (Thermo Scientific, USA), revealing a sufficient concentration for sequencing applications.

### 3.3 Whole-genome sequencing and assembly

The genome sequencing strategy employed a dual-technology approach for comprehensive coverage of the bacterial DNA. Short-read sequencing was conducted by first preparing a paired-end library using DNBSEQ (DNA Nanoball Sequencing) protocols as per manufacturer's guidelines (Beijing Genomics Institute, China). The library was subsequently sequenced on a BGISEQ-500 platform, which produced 150 base-pair paired-end reads. For the long-read component, Macrogen (Seoul, South Korea) prepared and sequenced a carefully size-selected SMRTbell library on their PacBio Revio system (Pacific Biosciences, USA). The resulting data were subjected to specialised quality control; short reads with fastp v0.23.4 [12] and long reads with fastplong v0.2.0 [13] to remove sequencing artefacts and ensure data integrity. These complementary datasets were then integrated through a hybrid assembly approach using Unicycler v0.5.1 [14], combining the accuracy of short reads with the spanning power of long reads. The quality of this genomic reconstruction was assessed using QUAST v5.2.0 [15] analysis, which evaluated assembly statistics and contiguity metrics.

### 3.4 Taxonomic identification of strains

The genomic quality of strain AMFE023/7 was rigorously assessed using CheckM2 v1.0.1 [16], which evaluated completeness, contamination, and heterogeneity parameters. A circular genome map of the AMFE033/7 strain was generated using Bakta v1.10.4 [17]. The taxonomic placement was determined via digital DNA-DNA hybridisation (dDDH) analysis using the Type (Strain) Genome Server (TYGS) [18]. The proposed platform facilitates comprehensive genome-based comparisons between strain AMFE023/7 and reference genomes from taxonomically related *Bacillus* species. Additionally, a phylogenomic tree was constructed using the TYGS platform with whole-genome sequences of type strains and default parameters to visualise evolutionary relationships, confirming the taxonomic position of the AMFE023/7 isolate.

### 3.5 Genome annotation and functional analysis

The *B. subtilis* AMFE023/7 genome sequence was annotated using the NCBI Prokaryotic Genome Annotation Pipeline (PGAP) [19], which provides comprehensive identification of coding sequences, RNA genes, and pseudogenes. To identify potential bioactive compounds, the genome was analysed using antiSMASH v7.1.0 [20], which identified several secondary metabolite biosynthetic gene clusters. The antibiotic biosynthesis pathway reconstruction was performed using the KEGG Automatic Annotation Server (KAAS). This analysis mapped the protein sequences of the strain AMFE023/7 against the KEGG database using the GHOSTX search algorithm. The bi-directional best hit (BBH) approach using a curated prokaryotic reference set revealed complete pathways for the biosynthesis of antimicrobial compounds [21].

## Limitations

The biosynthesis pathways of antibiotics and bioactive compounds identified in this research remain theoretical constructs based on computational predictions. Without experimental verification, these predicted pathways should be interpreted cautiously because they may not fully reflect the actual biochemical processes occurring within *B. subtilis* AMFE023/7. Future work involving functional genomics and biochemical analyses would be valuable to validate these in silico predictions and establish their true biological significance.

## Ethics Statement

This research excluded human or animal subjects; therefore, ethical approval was not required. The authors confirm that this manuscript contains original work that has not been previously published or submitted elsewhere.

## CRediT authorship contribution statement

**Montri Yasawong:** Methodology, Data curation, Writing – original draft, Writing – review & editing. **Thunwarat Songngamsuk:** Methodology, Data curation. **Manassanan Phatcharaharikarn:** Methodology, Data curation. **Parweenuch Santaweesuk:** Methodology. **Prapimpun Wongchitrat:** Methodology, Data curation. **Prasit Palittapongarnpim:** Methodology, Data curation. **Wongwarut Boonyanugomol:** Methodology, Data curation. **Arunee Thong-on:** Methodology. **Sasalux Kaewbutra:** Methodology. **Kamolchanok Rukseree:** Conceptualization, Data curation, Supervision, Writing – original draft, Writing – review & editing.

## Data Availability

Original dataComplete genome sequence data describing the biosynthetic potential of kanosamine and bacilysin in Bacillus subtilis AMFE023/7 from Thai fermented fish (Pla Ra) (Original data) (GenBank).

## References

[1] Goris J., Konstantinidis K.T., Klappenbach J.A., Coenye T., Vandamme P., Tiedje J.M. (2007). DNA-DNA hybridization values and their relationship to whole-genome sequence similarities. Int. J. Syst. Evol. Microbiol..

[2] Meena K.R., Kanwar S.S. (2015). Lipopeptides as the antifungal and antibacterial agents: applications in food safety and therapeutics. Biomed Res. Int..

[3] Wang T., Liang Y., Wu M., Chen Z., Lin J., Yang L. (2015). Natural products from *Bacillus subtilis* with antimicrobial properties. Chin. J. Chem. Eng..

[4] Sutyak K.E., Wirawan R.E., Aroutcheva A.A., Chikindas M.L. (2008). Isolation of the *Bacillus subtilis* antimicrobial peptide subtilosin from the dairy product-derived *Bacillus amyloliquefaciens*. J. Appl. Microbiol..

[5] Barbosa J., Caetano T., Mendo S. (2015). Class I and class II lanthipeptides produced by *Bacillus* spp. J. Nat. Prod..

[6] Dertz E.A., Xu J., Stintzi A., Raymond K.N. (2006). Bacillibactin-mediated iron transport in *Bacillus subtilis*. J. Am. Chem. Soc..

[7] Angelini L.L., Dos Santos R.A.C., Fox G., Paruthiyil S., Gozzi K., Shemesh M., Chai Y. (2023). Pulcherrimin protects *Bacillus subtilis* against oxidative stress during biofilm development. NPJ Biofilms Microbiomes.

[8] Kenig M., Vandamme E., Abraham E.P. (1976). The mode of action of bacilysin and anticapsin and biochemical properties of bacilysin-resistant mutants. J. Gen. Microbiol..

[9] Nannan C., Vu H.Q., Gillis A., Caulier S., Nguyen T.T.T., Mahillon J., the Bacilysin within (2021). *Bacillus subtilis* group: gene prevalence versus antagonistic activity against gram-negative foodborne pathogens. J. Biotechnol..

[10] Patel P.S., Huang S., Fisher S., Pirnik D., Aklonis C., Dean L., Meyers E., Fernandes P., Mayerl F., Bacillaene a novel inhibitor of procaryotic protein synthesis produced by (1995). *Bacillus subtilis*: production, taxonomy, isolation, physico-chemical characterization and biological activity. J. Antibiot..

[11] Astrup T., Mullertz S. (1952). The fibrin plate method for estimating fibrinolytic activity. Arch. Biochem. Biophys..

[12] Chen S., Zhou Y., Chen Y., Gu J. (2018). fastp: an ultra-fast all-in-one FASTQ pre-processor. Bioinformatics.

[13] Chen S. (2023). Ultrafast one-pass FASTQ data preprocessing, quality control, and deduplication using fastp. Imeta.

[14] Wick R.R., Judd L.M., Gorrie C.L., Holt K.E. (2017). Unicycler: resolving bacterial genome assemblies from short and long sequencing reads. PLoS Comput. Biol..

[15] Mikheenko A., Prjibelski A., Saveliev V., Antipov D., Gurevich A. (2018). Versatile genome assembly evaluation with QUAST-LG. Bioinformatics.

[16] Chklovski A., Parks D.H., Woodcroft B.J., Tyson G.W. (2023). CheckM2: a rapid, scalable and accurate tool for assessing microbial genome quality using machine learning. Nat. Methods..

[17] Schwengers O., Jelonek L., Dieckmann M.A., Beyvers S., Blom J., Goesmann A. (2021). Bakta: rapid and standardized annotation of bacterial genomes via alignment-free sequence identification. Microb. Genom..

[18] Meier-Kolthoff J.P., Göker M. (2019). TYGS is an automated high-throughput platform for state-of-the-art genome-based taxonomy. Nat. Commun..

[19] Tatusova T., DiCuccio M., Badretdin A., Chetvernin V., Nawrocki E.P., Zaslavsky L., Lomsadze A., Pruitt K.D., Borodovsky M., Ostell J. (2016). NCBI prokaryotic genome annotation pipeline. Nucleic Acids Res..

[20] Blin K., Shaw S., Augustijn H.E., Reitz Z.L., Biermann F., Alanjary M., Fetter A., Terlouw B.R., Metcalf W.W., Helfrich E.J.N., van Wezel G.P., Medema M.H., Weber T. (2023). antiSMASH 7.0: new and improved predictions for detection, regulation, chemical structures and visualization. Nucleic Acids Res..

[21] Moriya Y., Itoh M., Okuda S., Yoshizawa A.C., Kanehisa M. (2007). KAAS: an automatic genome annotation and pathway reconstruction server. Nucleic Acids Res..

